# Chemotherapy for prostate cancer: small steps or leaps and bounds? No huzzahs just yet!

**DOI:** 10.1038/sj.bjc.6602157

**Published:** 2004-09-10

**Authors:** D Raghavan

**Affiliations:** 1 Cleveland Clinic Taussig Cancer Center, 9500 Euclid Avenue, R35, Cleveland OH, 44195, USA

Prostate cancer has become a major public health issue. The application of screening technology incorporating digital rectal examination, measurement of PSA and use of transrectal ultrasonography has identified a huge reservoir of previously undetected cases. For a short time, this gave the semblance of an ‘epidemic’, although it soon became clear that this was not the case and only reflected the reservoir effect. Despite the absence of definitive, structured proof of the benefit of screening for prostate cancer, politicians, health authorities, advocacy groups and innocent bystanders have weighed into the debate on the benefits of screening and early intervention (Raghavan, 1997). This has focused attention on all aspects of prostate cancer care, including the utility of cytotoxic chemotherapy (Beer and Raghavan, 2000).

The Minireview by Canil and Tannock has accurately summarised what we know about the benefits and drawbacks of chemotherapy for prostate cancer (Canil and Tannock, 2004). For locally advanced disease, several provocative phase I–II trials have suggested that there may be a biological effect from the early use of cytotoxics, either prior to definitive treatment or as classical adjuvant therapy. In truth, a larger biological impact on the outcome of treatment for locally advanced (stage C) disease is seen from hormonal therapy, especially when delivered over a protracted period of 1–3 years (Bolla *et al*, 2002). An important randomised trial (Southwest Oncology Group (SWOG) 9921) is currently assessing the role of adjuvant hormonal therapy plus mitoxantrone, compared to adjuvant hormones alone, for locally extensive prostate cancer after radical prostatectomy. As Canil and Tannock note, there is no role for ‘routine’ neoadjuvant or adjuvant cytotoxics for locally advanced prostate cancer unless in a structured clinical trial.

For metastatic disease, at the time that the review was written, there was no definitive published proof of a survival benefit from chemotherapy. The authors noted that chemotherapy could improve quality of life for patients with metastatic hormone refractory disease. In the study reported by Tannock *et al* (1996), there was a dramatic improvement in the quality of life and cost of treatment (Bloomfield *et al*, 1998), but no obvious survival benefit gained from early institution of mitoxantrone chemotherapy. Importantly, however, this study allowed crossover, and patients treated only with prednisone initially were allowed to receive subsequent chemotherapy upon relapse, thus potentially vitiating any survival benefit. In my view, the trial reported by Tannock *et al* suggested that it may be safe to use conservative means initially prior to implementing cytotoxic chemotherapy; that is, there was no obvious survival benefit from early chemotherapy compared to elective chemotherapy after first subsequent relapse.

Since the submission of this Minireview, the results of two trials mentioned by the authors have entered the public domain, having been presented at the Annual Scientific Meeting of the American Society of Clinical Oncology. Eisenberger *et al* (2004) presented the results of a study sponsored by Aventis Pharmaceuticals, TAX 327, which compared a schedule of 3-weekly taxotere *vs* weekly taxotere *vs* 3-weekly mitoxantrone, each agent being administered with continuous oral prednisone. For the first time, this trial showed a statistically significant survival benefit achieved from 3-weekly taxotere compared to 3-weekly mitoxantrone. Consistent findings were identified with respect to symptomatic improvement, PSA reduction and objective tumour response. Of importance, the difference in the median survival was only 2 months, and there was no dramatic difference in outcomes beyond 2 years. Similarly, Petrylak *et al*, reporting results of a North American Intergroup trial led by the Southwest Oncology Group (trial SWOG 9921), demonstrated a statistically significant difference between mitoxantrone–prednisone and taxotere–estramustine. Once again, the difference in the median survival was only 2 months and there was no obvious long-term impact. Nonetheless, these studies do show a clear role for cytotoxic chemotherapy that includes improvement in survival, objective and subjective tumour response and quality of life.

One of the complex issues that remain unresolved is our inability to measure quality of life optimally for patients with prostate cancer. The studies cited above (Tannock *et al*, 1996; Eisenberger *et al*, 2004; Petrylak *et al*, 2004) all demonstrate surprising discordance between response, measured quality of life, assessment of pain on structured quantification scales and long-term outcome. This may be due to the impact of toxicity; for example, it is clear that taxotere causes more side effects than does mitoxantrone, and this may contribute to this discrepancy. Alternatively, it may reflect the oft underestimated impact of castration, with a range of physical, emotional and behavioural costs.

What is quite clear is that we are making slow progress in evolving treatment of prostate cancer…but it IS progress. Slowly the median survival is improving, notwithstanding the impact of stage migration. We are evolving strategies for measuring quality of life, and there is a clear emphasis on the attempt to reduce toxicity of treatment. At present, it appears that taxotere has become a new standard initial chemotherapy for metastatic prostate cancer, but it is clearly an imperfect standard that does not merit the loud huzzahs that will clearly follow. Mitoxantrone also remains a useful agent, and it is really not clear whether a huge gain is achieved by the use of one agent first. In fact, it may well be that there is still a place for the vanishing paradigm of old-fashioned clinical skill and judgment. For example, the patient with multiple medical issues and slowly evolving, symptomatic disease may well be treated more successfully by a gentler induction regimen.

The final issue is to understand why progress in prostate cancer has been so much slower than in cancer of the breast. It has been postulated that prostate cancer is more heterogeneous and innately drug resistant, that it afflicts older and less robust patients, or that the impact of neuroendocrine cells confounds the assessment of outcome. To me, the answer is relatively simple: failure of many physicians to encourage their patients to enter structured clinical trials that reflect sensible clinical questions and well-defined end points or failure of patients to accept the concept that uncertainties in medicine are often best resolved through clinical trials rather than via rash empiricism.
